# Impact of Implant Surface and Smoking on Peri-Implant Human Bone: What we Learned from The Last 20 Years?

**DOI:** 10.1590/0103-6440202406115

**Published:** 2024-10-25

**Authors:** Jamil A. Shibli, Marcio C. Formiga, Giselle A. Elias, Carlos F Mourão, Leonardo P. Faverani, João G. S. Souza, Giovanna Iezzi, Adriano Piattelli

**Affiliations:** 1 Department of Periodontology, Dental Research Division, Guarulhos University, Guarulhos, SP, Brazil; 2 Department of Periodontology, Tufts University School of Dental Medicine, Boston, MA, USA; 3 Division of Oral and Maxillofacial Surgery and Implantology, Department of Diagnosis and Surgery, School of Dentistry, São Paulo State University (UNESP), Araçatuba, São Paulo, Brazil.; 4 Department of Medical, Oral and Biotechnological Sciences, "G. d'Annunzio"University of Chieti-Pescara, Chieti, Italy.; 5 School of Dentistry, Saint Camillus International University of Health and Medical Sciences, Rome, Italy.

**Keywords:** Dental implants, osseointegration, implant surface topography, smoking, histology

## Abstract

The present review summarizes the findings from human histological studies conducted over the past 20 years at the University of Guarulhos, Brazil, examining the impact of various implant surface topographies and smoking on peri-implant bone response. Seven different implant surfaces were evaluated in 90 partially or completely edentulous individuals using a total of 123 micro-implants. Histometric parameters, including bone-implant contact (BIC%), bone area within the threads (BA%), and bone density (BD), were assessed after an 8-week healing period. Scanning electron microscopy (SEM) and X-ray diffraction (XRD) analyses were also performed. Results showed that treated surfaces, regardless of the treatment type, consistently demonstrated better histometric outcomes compared to machined surfaces. Anodized surfaces and those subjected to airborne particle abrasion, followed by acid etching, exhibited higher BIC% values than machined surfaces in smoker patients. Smoking reduced BIC% around anodized implants. The presence of inflammatory cells was observed adjacent to the peri-implant soft tissue on some treated surfaces. In conclusion, implant surface topography significantly influences early bone response under unloaded conditions, with treated surfaces promoting better human bone tissue response than machined surfaces. However, smoking negatively impacts peri-implant bone healing, emphasizing the importance of smoking cessation for optimal osseointegration.

## Resumo

A presente revisão resume os achados dos estudos histológicos avaliando o impacto de diferentes superfícies de implantes sobre os tecidos peri-implantares humanos nos últimos 20 anos na Universidade Guarulhos (UnG), Brasil. Sete diferentes superfícies de implantes foram avaliadas em 90 indivíduos utilizando um total de 123 micro-implantes. Parâmetros histométricos incluindo contato osso-implante (BIC%), área óssea entre as espiras (BA%), e densidade óssea (BD%) foram avaliados após 8 semanas de cicatrização. Microscopia eletrônica de varredura (SEM) e difração de raios X (XRD) também foram realizados. Os resultados mostraram que independente do tipo de tratamento de superfície, os parâmetros histométricos foram consistentemente melhores que os achados nas superfícies lisas. Superfícies anodizadas e as tratadas com jatos de partículas abrasivas seguidos por ácidos, exibiram maiores índices de BIC% que superfícies lisas em pacientes fumantes. O fumo reduziu o percentual de BIC% ao redor de implantes anodizados. A presença de células inflamatórias foi observada adjacente aos tecidos moles peri-implantares de algumas superfícies. Em suma, a topografia de implantes aumentou significantemente a resposta precoce da osseointegração sob condições de ausência de carga oclusal, promovendo melhores resultados quando comparados a superfície lisa. Entretanto o fumo influenciou negativamente a cicatrização óssea, enfatizando a importância de cessar o fumo para uma melhor osseointegração.

**Figure:**
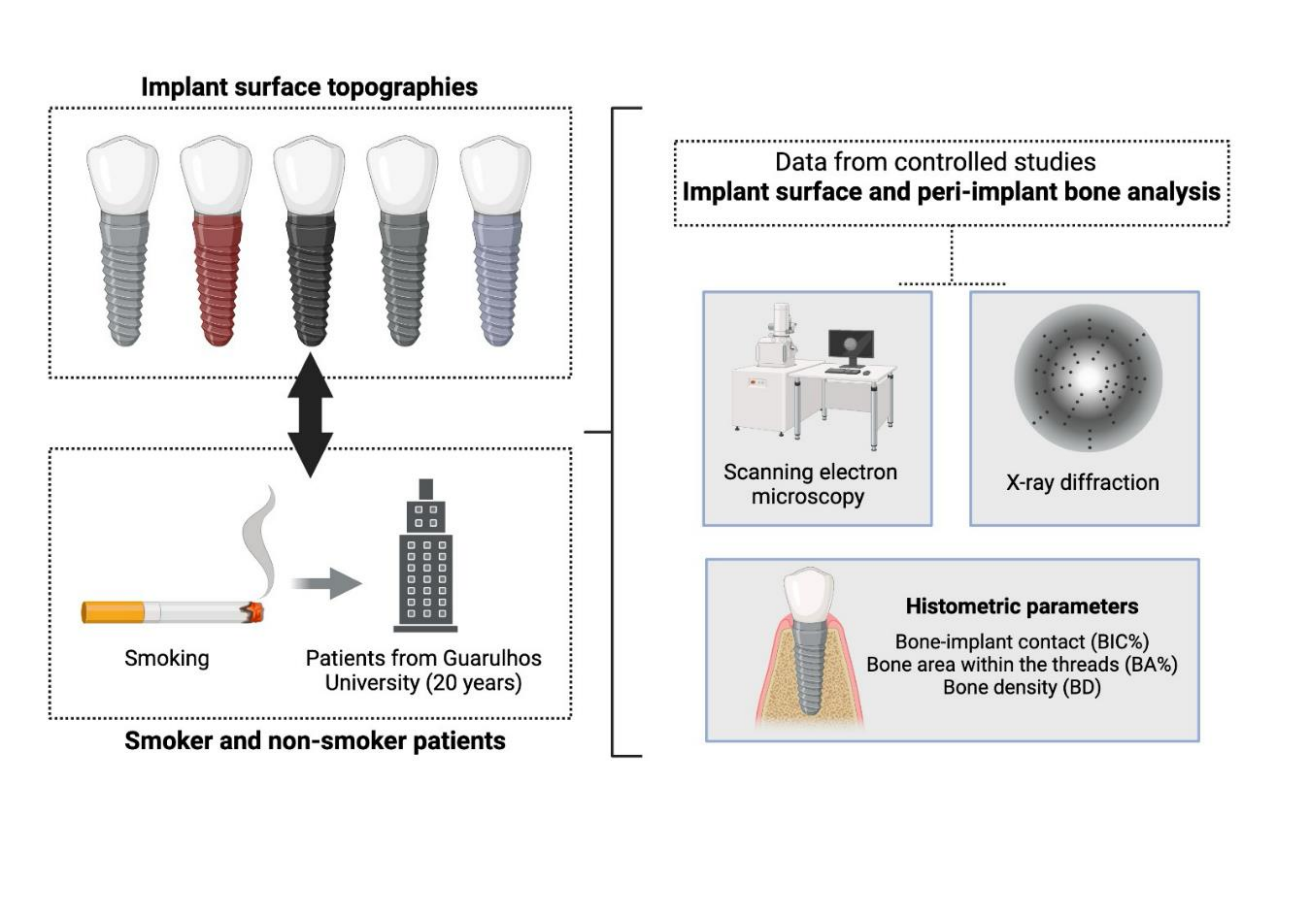

## Introduction

The use of osseointegrated implants in the rehabilitation of partially or completely edentulous individuals has increased considerably over the last few years due to their high success rates ^1^ ^,^ ^2^. Osseointegration, also known as functional ankylosis, refers to the percentage of bone tissue in contact with the implant surface while in function under an optical microscope. Therefore, the functional and structural unit of Implant Dentistry was based on the concept of bone-implant contact and, consequently, all the events and factors that influenced this process such as surgical trauma, biocompatibility of the implant surface, macro- and microstructures as well as the comprehension of the wound healing ^1^ ^,^ ^2^ ^,^ ^3^ ^,^ ^4^.

In the last 30 years, factors related to dental implants (implant microgeometry, type, and chemical composition of the implant surface) and to the host (local and systemic factors) have been evaluated with a view to perfecting and developing technologies for the macro- and micro-structures ^5^ ^,^ ^6^. Among these factors, the implant surface has received the most attention ^7^. The implant microstructure influences the quantity and quality of bone-implant contact and, consequently, increases the long-term success of osseointegrated implants because, theoretically, the better the bone-implant contact, the longer will be the life expectancy of this implant. This idea is based on the concept that higher is the dental bone anchorage, better will be the maintenance of the peri-implant bone environment during the load conditions.

The characteristics of the implant surface influence the complex process of osseointegration in different ways. Previous studies ^8^ ^,^ ^9^ have shown that titanium's pragmatic biocompatibility ^5^ has advantages compared with other materials, such as the low inflammatory reaction between the cells adjacent to the commercially pure titanium cpTi during osseointegration process ^10^.

However, some studies have reported failures in the process of osseointegration ^11^ ^,^ ^12^. These failures were classified as early and late types. The early types were related to problems such as a lack of biocompatibility of the implant surface, infection in the receptor site, surgical trauma, or any other event that would make it impossible for the dental implant to osseointegrate, even before it received load or masticatory effort. The late failures were related to peri-implant infections either associated or not with occlusal overload ^13^ ^,^ ^14^.

Although these failures in osseointegration were associated with several factors, the most common was the difficulty in forming peri-implant bone tissue, particularly in areas of low-density bone (15-20) and among smokers ^21^ ^,^ ^22^.

However, other studies ^23^ ^,^ ^24^ ^,^ ^25^ almost simultaneously evaluated the immediate and early activation of osseointegrated implants with machined and rough surfaces, resulting in a reduction in healing time and activation of osseointegrated implants. Clinical ^26^ and histological ^27^ studies showed far more promising data about the immediate activation and loading of the implants, with success rates comparable with those of implants loaded after a period ranging from 4 to 6 months, as proposed by Adell et al., ^28^ and Albrektsson et al., ^29^. These time intervals (4 months in the mandible and 6 months of healing in the maxilla) were proposed to ensure that osseointegrated Ticp implants would be apt to receive masticatory forces. These osseointegrated implants would only support these masticatory forces if they had reached a level of osseointegration of at least 50% ^30^ ^,^ ^31^ ^,^ ^32^.

In this context, Implant Dentistry was being implemented in clinical practice, encouraging new considerations related to implant surfaces in both in vitro and in vivo studies ^33^ ^,^ ^34^. Various systematic reviews ^6^ ^,^ ^7^ ^,^ ^35^ ^,^ ^36^ have proposed that osseointegrated implants with treated or rough surfaces could receive masticatory loading in a shorter period with success rates that were higher than the ones compared with the rates of Ticp surfaces.

## Influence of different types of implant surfaces

Clinical ^37^ and histological studies ^30^ ^,^ ^38^ have proposed that osseointegrated implants with treated or rough surfaces could receive masticatory loading in a shorter period than was previously recommended by Adell et al. ^28^ and Albrektsson et al. ^29^. These clinical and in vitro studies evaluated the percentage of osseointegration, seeking to improve the type of surface of the implants.

In addition to the type of microstructure, the percentage of osseointegration depends on the surgical technique, the systemic conditions of the individual, and the quantity and quality of bone tissue ^37^. The availability of bone is reduced after tooth loss and resorption of alveolar bone tissue, mainly in posterior regions of the maxillary ^16^. The certain degree of roughness implant surface topography facilitates the blood clot adhesion and consequently allow the direct bone formation or contact osseointegration, increasing the bone-to-implant contact percentage. The rate of bone-implant contact has been reported to be between 25 and 65% for commercially pure titanium (cpTI), also called machined surface and from 45 to 70% for roughness titanium surfaces for studies in both animals ^39^ ^,^ ^40^ and humans ^19^ ^,^ ^38^. Moreover, these investigations have shown that osseointegrable dental implants with machined surfaces, placed in type IV bone (posterior region of the maxilla and grafted areas) have high rates of failures compared with other areas with greater bone density ^15^ ^,^ ^41^.

Consequently, rough or textured implant surfaces may facilitate healing by increasing the percentage of bone-implant contact in areas with low density bone ^18^ ^,^ ^42^ ^,^ ^43^. The success of dental implants relies on their properties, including chemical, physical, mechanical, and surface characteristics. These different properties interacted among them, influencing cell activity around the implant surface ^35^. Based on these data, various studies have investigated different implant surfaces, obtained by means of addition (coating with titanium plasma, hydroxyapatite) or subtraction techniques (airborne particle abrasion with different types of materials such as titanium or aluminum oxide), as well as treatments with acids, and laser preparation ^7^.

The properties of these new surfaces influence the bone cells that migrate and proliferate in the surgical site instrumented for insertion of the implant that has better bone-implant contact rates due to the increased contact area of the implant surface ^6^ ^,^ ^33^. As a complement, this surface roughness provides a configuration that improves retention of the blood clot, stimulates and facilitates the process of osseointegration, and consequently allows these implants to be submitted to prosthetic loading after a shorter healing period ^30^ ^,^ ^44^.

### Histological Studies in Humans

The use of animal models to test the influence of different implant surface types has some limitations inherent to each experimental model, such as the type of occlusion, healing time and cell repair of the bone tissue, differences in adsorption of both cellular and protein components, frequently in addition to showing evidence of the biological event, however, without being reproducible in human beings ^19^ ^,^ ^38^ ^,^ ^42^ ^,^ ^45^ ^,^ ^46^. This is the reason why some authors have proposed an evaluation of the percentage of osseointegration in the human maxilla using osseointegrated implants with the same types of surfaces as those commercially available but with reduced sizes ^30^ ^,^ ^38^ ^,^ ^42^ ^,^ ^45^ ^,^ ^47^ ^,^ ^48^.

Although scarce, some histological studies have systematically investigated the process of osseointegration in human maxillae. Two different studies ^38^ ^,^ ^42^ investigated the effect of different types of implant surfaces on human maxillae. Ivanoff et al. ^42^ made a histological assessment of osseointegration by using micro-implants with the titanium surface airborne particle abraded with titanium oxide (TiO_2_) particles and Ticp surface. Twenty-seven patients received two micro-implants each: one Test (TiO2) and one Control (cpTi). The micro-implants were removed after a mean healing period of 6.3 months for the maxilla and 3.9 months for the mandible. Histomorphometric evaluation showed a significantly higher bone-implant contact value for the airborne particle-abraded implants in both the mandible and maxilla.

With the same methodology, Ivanoff et al. ^38^ made a histological assessment of the bone response to a micro-implant with an anodized surface and a smooth surface, in humans. After 6.6 months in the maxilla and 3.5 in the mandible, the micro-implants were removed and submitted to histomorphometric evaluation. The anodized surface showed a higher percentage of osseointegration and bone filling between the implant threads.

Trisi et al. ^32^ evaluated the influence of two different types of surfaces prepared on the same micro-implant inserted into medullary bone. Each micro-implant was inserted in the posterior region of the maxilla in 11 partly edentulous patients and removed after a time interval of 60 days. Using polarized light and a conventional optical microscope, the authors observed a percentage of bone-implant contact of 19.00%±14.68% and 47.81%±14.01% for the machined surface and airborne abraded surface/treated with acids, respectively. As a complement, using the image superimposition technique, the authors also observed that the machined surface showed a reduction of 44.7% in bone-implant contact.

Orsini et al. ^49^ evaluated the influence of implants with calcium-phosphate (CaP) coating on a nanometric scale, in which these particles were visualized by scanning electron microscopy at 50x magnification. The micro-implants were divided into a Control Group (composed of micro-implants with airborne abraded surfaces and treated with acids) and a Test Group (consisting of implants with nano-roughness surface topography). Fifteen patients received the micro-implants in the posterior region of the maxilla: nine patients received implants from both groups, five patients received only implants from one group, and one patient received 4 micro-implants (2 from each group) totaling 32 micro-implants (16 from the test group and 16 from the control group). The implants were removed after 8 weeks of healing, and histomorphometric evaluation was performed. The percentage of bone-implant contact ranged between 0 and 65% for the micro-implants, with the mean values being 19% and 32.2% for the Control and Test Groups, respectively, suggesting that the surface into which CaP had been incorporated, could reduce the healing time and improve the success rates in cases of early occlusal loading.

Finally, Lang et al. ^46^ evaluated the initial stages of healing (at time intervals of 7, 14, 21, 28 and 42 days) for micro-implants with hydrophilic and hydrophobic types of surfaces. Differently from the previously cited studies in which the implants were inserted into edentulous spaces, the authors inserted the micro-implants in retromolar regions of 28 volunteers (students and employees of the institution). In 21 patients, micro-implants were inserted bilaterally (one micro-implant of each group). After removing the implants in the different time intervals, histometric evaluation was performed in only 30 of the 49 implants inserted. The micro-implants removed in the initial time intervals were found to be difficult to analyze, because they frequently showed no bone formation, that would make it possible to perform a histometric analysis, so that only histological analyses were performed. The percentage of bone-implant contact gradually increased for both types of surfaces, however, it was only at 28 days that the hydrophilic surface showed significantly higher differences than the hydrophobic group (p<0.05). Whereas at 42 days, both types showed the same 62% of bone-implant contact (p>0.05). Although the main objective of this study was to evaluate bone-implant contact, the authors also concluded that the rate of osseointegration in humans was slower when compared with the same study conducted in an animal model.

### Smoking

In addition to the factors related to osseointegrated implants, mainly the microstructure, those inherent to the host were also able to influence the rate of osseointegration and, consequently, the long-term success of the implant-supported restorations ^50^. Factors such as diabetes, smoking and osteoporosis have been extensively evaluated by various researchers, using histological models in animals, and some clinical studies in humans. These factors have the influence on the bone tissue healing process in common.

Smoking has been reported to be a risk factor for periodontitis and for peri-implantitis ^51^. Other studies have correlated smoking with early loss of implants, increase in marginal bone loss, and problems with soft tissue healing ^52^. Some studies using animal models ^53^ ^,^ ^54^ that evaluated the healing of alveoli in humans ^55^ had shown the deleterious effect of inhaling cigarette smoke and nicotine on the apposition of bone tissue to the implant surface. These studies indicate that cigarettes influence the healing of peri-implant bone tissue by both the local effect (heat and smoke) and systemic effect of inhaling all the toxic substances present in cigarettes, thereby reducing the bone-implant contact and increasing the rate of implant losses before the installation of the prosthesis (early failures).

Cigarette smoke is composed of over 4.000 toxic substances that act directly systemically on the body. Substances such as nicotine, carbon monoxide, benzine aldehydes, and cyanides have deleterious effects on healing and cellular events related to apposition and bone cell turnover ^56^. Nicotine is a powerful vasoconstrictor that reduces the blood flow and nutrients to the surgical site, in addition to inhibiting the proliferation of fibroblasts, macrophages, and blood cells. Carbon monoxide diminishes the capacity of blood cells for transporting oxygen, consequently increasing the quantity of cyanide and leading to tissue hypoxia.

As a local complement, bone formation and regeneration are strictly related to angiogenesis, by the invasion of blood vessels and arteries into the surgical site undergoing the process of healing. The negative influence of cigarettes on the process of angiogenesis, development of leukocytes and on the levels and functions of some cytokines, such as for example, osteoprotegerin - when this is reduced, it diminishes the rate of bone apposition - which may in part, justify the results obtained in clinical studies that evaluated the low success rates and the higher level of marginal peri-implant bone loss in smoker patients ^50^ ^,^ ^52^.

### Human peri-implant tissue evaluation at the University of Guarulhos

In the last twenty years, the Department of Periodontology at Dental Research Division (UnG), Brazil, has systematically evaluated the impact of several implant surface topographies. Therefore, this narrative review evaluated all the controlled studies comparing different implant surface topographies either in smoker and non-smokers subjects developed in the department; case reports, systematic reviews and narrative reviews were not included in this review.

All included studies used the methodology that place experimental micro-implants with different implant surface topography in human jaws. These micro-implants were removed after the initial stage of healing and that were not submitted to masticatory force (occlusal loading).

In detail, the studies described in this review were as follows:

- evaluate the influence of surfaces submitted to airborne particle abrasion with titanium oxide (TiO2) particles, and treated with the combination of nitric (HNO3) and hydrofluoric (HF) hydrofluoric acids ^43^ ^,^ ^57^, anodized surfaces ^19^ ^,^ ^58^, surfaces impregnated with bioceramics at the nanometer scale ^59^ and micro-implants produced with sintered titanium and treated with acids ^31^ ^,^ ^60^ ^,^ ^61^ on the percentage of bone-implant contact, bone density restricted to the area of the threads, and the density at a distance of 200 to 500 μm from the implant, after 8 weeks of bone tissue repair;

- evaluate the impact of the smoking habit on bone-implant contact of micro-implants

with anodized surfaces, removed after 8 weeks of healing ^62^; and - evaluate the impact of implant surfaces airborne particles abraded with aluminum oxide (Al_3_O_2_) particles and treated with nitric acid (HNO_3_) on the rate of bone contact in smoker patients ^63^.

A total of 90 partially or completely edentulous individuals (54 women and 36 men) (mean age 50.43±14.5 years) participated in this study. They were patients from the Implant Dentistry Clinic of Guarulhos University who needed oral rehabilitation in the posterior region of both the mandible and maxilla by means of implant-supported dental prostheses ^19^ ^,^ ^31^ ^,^ ^43^ ^,^ ^57^ ^,^ ^58^ ^,^ ^59^ ^,^ ^60^ ^,^ ^61^ ^,^ ^62^ ^,^ ^63^.

Individuals were included who had at least one edentulous area in the posterior region (of the maxilla or mandible) with bone available for the insertion of conventional osseointegrated implants and the micro-implants. However, individuals who had chronic diseases such as rheumatoid arthritis and diabetes, chronic alcoholics, use of glucocorticoid or other immunosuppressant drugs; moderate or advanced periodontitis (characterized as the presence of probing depth and clinical attachment level > 5mm and bleeding on probing in over 30% of the sites); diseases of the oral mucosa; history of radiotherapy in the region of the head and neck, smokers and ex-smokers were excluded from the studies ^19^ ^,^ ^31^ ^,^ ^42^ ^,^ ^56^ ^,^ ^57^ ^,^ ^58^ ^,^ ^60^. In the other two studies ^61^ ^,^ ^62^, smoker patients were included, characterized as being those who smoked over 10 cigarettes (in paper with a filter) per day for over 5 years. In these studies ^61^ ^,^ ^62^, ex-smokers were also excluded from the population sample to avoid bias in the bone tissue response. Therefore, only smokers and nonsmokers were included.

The individuals included in the population sample were informed about the surgical procedures and removal of biopsies, to which they would be submitted and signed a Term of Free and Informed Consent, previously approved by the Research Ethics Committee of the University of Guarulhos.

### Experimental Design and micro-implants

**Figure 1:**
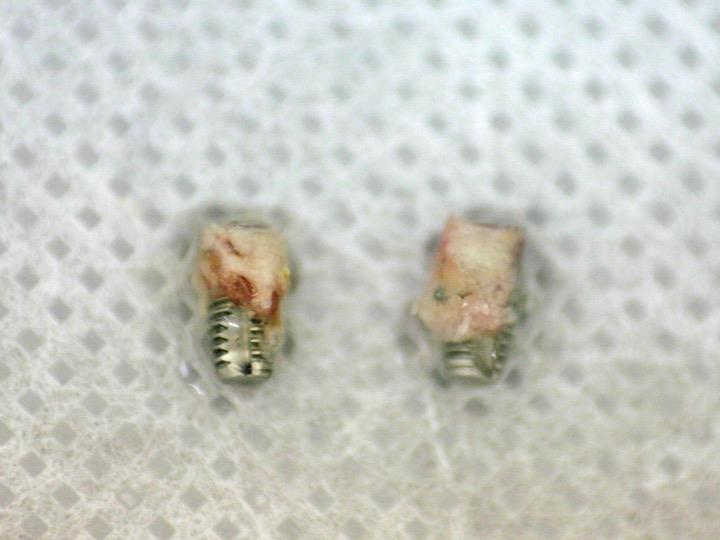
Detail of retrieved micro-implants (6mm long x 2mm diameter) from the maxilla. Note the difference in bone-surface density between the machined (left) and sandblasted acid-etched-surface (right) implants (Grassi et al. *J. Periodontol* 2006; License number 5800330837972)

The micro-implants were produced according to the model used by Ivanoff et al. ^42^. In total, 123 micro-implants with seven different types of surfaces were used in all the studies ^19^ ^,^ ^31^ ^,^ ^43^ ^,^ ^57^ ^,^ ^58^ ^,^ ^59^ ^,^ ^60^ ^,^ ^61^ ^,^ ^62^ ^,^ ^63^. The micro-implants used in some of the studies ^19^ ^,^ ^31^ ^,^ ^43^ ^,^ ^57^ ^,^ ^58^ ^,^ ^60^ ^,^ ^62^ measured 2.5 mm in diameter and 6mm long; in two studies ^59^ ^,^ ^61^ the micro-implants were 2.5 mm in diameter and 8 mm long, while the micro-implants of the last study ^63^ were 2.0 mm in diameter and 4 mm long (Figure 1).

Each individual received two micro-implants, of which one was Control and one Test (19,43,57-59,62), while the individuals in studies ^31^ ^,^ ^60^ ^,^ ^61^ ^,^ ^63^ received only one micro-implant. The micro-implants were inserted in the posterior region of the mandible or maxilla, always distal to the last conventional implant used for prosthetic rehabilitation.

The study sample size was based on the data of Ivanoff et al. ^42^ and Ivanoff et al. ^38^, in which, by means of difference and standard deviation of software (Power/Sample size Calculator-http://stat.ubc.ca/rollin/starts/ssize/n2.html), a power of over 80% was obtained in a sample larger than or equal to 10 individuals.

### Preparation of micro-implant surfaces

**Figure 2:**
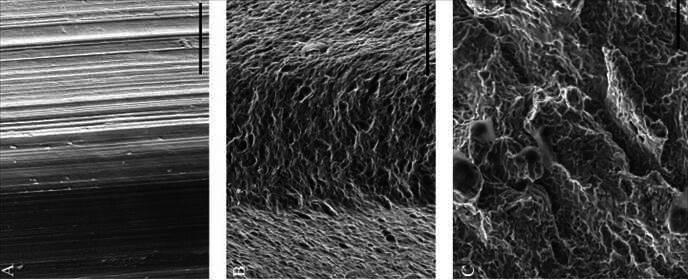
Scanning electron microphotograph of the (A) machined, (B) sandblasted acid-etched, and (C) direct laser fabrication surfaces (Barr 5-50um). (Shibli et al. *J Biomed Mat Res A* 2010; License number 5800330712013)

All micro-implants were prepared in accordance with the technical standards of each company and industry (Figure 2). The implants with smooth surfaces were prepared with Grade 4 titanium machined without any prior treatment (Conexão Sistema de Implantes, Aruja, São Paulo, SP and AS Technology, Titanium Fix, São José dos Campos, SP). The surfaces of implants used ^31^ ^,^ ^43^ ^,^ ^57^ were cpTi surfaces airborne particles abraded with titanium oxide (TiO_2_) particles, ranging between 25-100 μm in size, and afterward submerged in a combination of Nitric (HNO_3_) and Hydrofluoric (HF) acids, ultra-sonicated in an alkaline solution, and abundantly washed with distilled water (Conexão Sistema de Implantes, Aruja, São Paulo, SP). Although in another study ^63^, the surface of the implant was also airborne particle abraded and treated with acids, this micro-implant (AS Technology, Titanium Fix, São José dos Campos, SP) differed from the previous type due to the use of aluminum oxide (Al_3_O_2_) particles of 100 μm and by the treatment with HNO_3_.

The anodized surface was also prepared from the smooth surface, which, after treatment with acetone, was submerged in a solution prepared with HNO3 and HF and washed with distilled water ^19^ ^,^ ^58^. Immediately, the micro-implants were next anodized by means of a continuous electric current with electrolytes that had calcium glycerol phosphate/calcium acetate (C3H_7_CaO_6_P) in their composition. Then, they were abundantly washed with distilled water and dried (Conexão Sistema de Implantes, Aruja, São Paulo, SP).

The micro-implants used in one study ^59^ were submitted to a double acid attack or impregnation with calcium phosphate (CaP) at a nanometric scale - Ossean® (Intra-Lock International, Boca-Raton, Fl, USA). The implants sintered from titanium particles have previously been described by Mangano et al. ^44^. To sum up, Ti-6Al-4V particles between 25-45 μm (Leader, NOVAXA, Milan, Italy), were processed in an argon atmosphere using a Yb (Ytterbium) laser with a 250/250/215 mm3 fiber, using a wavelength of 1054 nm in continuous mode, with a power of 200 W at 7 m/s by means of a 0.1 mm spot. The micro-implants were sonicated in distilled water to remove residues inherent to sinterization and immersed in a mixture of NaOH (20 g/L) and H2O2 (20 g/L) at 80 ^o^C for 30 minutes. After further cleaning in distilled water, the micro-implants were again submerged in a 50% oxalic acid and 50% maleic acid solution.

### Placement of conventional osseointegrated and micro-implants

After preparing individualized surgical-prosthetic planning, the size of the conventional implant to be placed was selected based on the bone availability and implant localization in the dental arch. Surgical procedures for insertion of osseointegrated implants were performed as follows: antisepsis, local anesthesia, incision and divulsion of the mucoperiosteal flap, instrumentation of the surgical site and insertion of the osseointegrated implant that would support the implant-supported dental prosthesis. After the individuals received the conventional osseointegrated implants for prosthetic rehabilitation, the micro-implants were placed (see item *Experimental Design*).

The micro-implants were always placed distal to the last conventional osseointegrated implant. In articles ^19^ ^,^ ^43^ ^,^ ^57^ ^,^ ^58^ ^,^ ^63^, if an individual had a bilateral edentulous area, a micro-implant was placed on each side; if an individual had only one edentulous area, the 29 micro-implants were placed side by side, in the portion most distal to the last implant. In articles (31,59-62), only one micro-implant was placed in each patient.

The surgical sites for insertion of the micro-implants were prepared with 1.8 mm burs in diameter, in areas of type IV bone; and 2.2 mm burs in diameter in areas with type I and II bone density. If there was no primary stability during the insertion of the micro-implants by means of hexagonal digital keys of 1.6 mm, a second surgical site was prepared.

During all the procedures of surgical site preparation and placement of micro-implants, abundant irrigation with sterile solution was conducted. The flaps were sutured with interrupted sutures, and the micro-implants were submerged. All patients received an antibiotic regimen consisting of Clindamycin 300mg, 3x a day/7 days, and potassium diclofenac 50mg, 3x day/5 days to prevent post-surgical infections, inflammatory reactions, and pain control. In addition, the use of a mouthwash with 0.12% chlorhexidine digluconate solution, twice a day, for 14 days was recommended, to control biofilm accumulation. The sutures were removed after 10 days.

### Removal of the implants and histological processing

After 8 weeks, during surgery for reentry the conventional osseointegrated implants ^31^ ^,^ ^63^, the micro-implants, and the circumjacent bone tissue were removed by means of a trephine bur of 3.25 mm in diameter and placed in 4% neutral Formaldehyde solution.

The biopsies were processed, and thin sections of the bone-implant block were obtained (Precise 1 Automated System®, Assing, Rome, Italy), as described by Piattelli et al. ^65^. The biopsies were dehydrated by means of washing in increasing concentrations of ethanol alcohol (60-100%) and infiltrated with glycolmethacrylic resin (Technovit® 7200 VLC, Kulzer, Wehrheim, Germany) in increasing concentrations.

After this the block was embedded in 100% glycerol methacrylate. The resin blocks containing the biopsies were then sectioned along the largest axis of the implant. Using a high precision diamond-coated disc, a 150 μm thick section was obtained. This section was submitted to a micro-wear system using water abrasive paper, which resulted in a section with a thickness of approximately 30 μm.

One or two slides, depending on the size of the biopsy, were obtained for each implant. Each section was stained with basic fuchsin and toluidine blue. For evaluation of bone tissue repair close to the implant, in addition to the histological analysis, the following histometric parameters were analyzed:

1. . *The percentage of bone-implant contact (BIC%):* measured around the entire perimeter of the plant: calculated by direct contact between the bone-implant, multiplied by 100, divided by the total perimeter of the implant ^19^ ^,^ ^31^ ^,^ ^43^ ^,^ ^57^ ^,^ ^58^ ^,^ ^59^ ^,^ ^60^ ^,^ ^61^ ^,^ ^62^ ^,^ ^63^;
2. . *Bone area within the implant threads (BA%):* calculated by the area filled with bone tissue within the threads, multiplied by 100 and divided by the total area of the micro-implant thread ^19^ ^,^ ^31^ ^,^ ^43^ ^,^ ^57^ ^,^ ^58^ ^,^ ^59^ ^,^ ^60^ ^,^ ^61^ ^,^ ^62^ ^,^ ^63^;
3. . *Bone Density (BD):* measurement of the distance between 500 μm to 200 μm (only for articles based on the micro-implant thread) calculated by the area filled with bone tissue in this region, multiplied by 100 and divided by the total area ^19^ ^,^ ^43^ ^,^ ^57^ ^,^ ^58^ ^,^ ^62^;
4. . *Osteocyte Index (Oi):* obtained by using the equation Oi=NOt/AB in which NOt is the total quantity of osteocytes present in an area of 50 μm on the implant surface, visualized at 200x magnification, and AB is the area expressed in μm2 ^59^.

The previously calibrated examiners of each study, who had no knowledge of the surfaces (blinded), made all the measurements at two different time intervals, using an optical microscope (Laborlux S®, Leitz, Wetzlar, Germany) with a 4.0/10 X magnification lens, by means of which the images were selected and sent to a microcomputer by a video camera (3CCD®, JVC KY-F55B, Milan, Italy), attached to the optical microscope. The values were determined by using an image analyzing software program (Image-Pro Plus® 4.5, Media Cybernetics Inc Immagini & Computer Snc, Milan, Italy). For the Oi, additional software programs were used: (Adobe Photoshop CS, version 8.0.1, Adobe Systems, Beaverton, OR, USA and Image J1.32j, Wayne Rasband, National Institutes of Health, Bethesda, MD, USA) to help with cell counts and calibrate the histological images, respectively.

### Analysis by Scanning Electron Microscopy (SEM) and X-ray diffraction (XRD)

The biopsies were removed from the formaldehyde solution and washed with sodium cacodylate buffer solution, dehydrated in increasing concentrations of ethanol and hexamethyldisilazane (HMDS), and sputter-coated with gold for evaluation by scanning electron microscopy (FEI XL-30 FEG, Philips, Toronto, Canada) operated at voltages between 7 and 15kV ^61^. The images were obtained in TIFF format in dimensions of 1424 x 968 pixels. The X-ray analyses were performed with the same equipment, in which the mapped areas were 512 x 512 BMP. The images of the same area obtained by SEM and XRD were combined by means of a software program (Adobe Photoshop CS, version 8.0.1, Adobe Systems, Beaverton, OR, USA) and evaluated by an examiner to determine the presence of Ca, P, and Ti.

### Statistical Analysis

The mean and standard deviation of histometric variables were calculated for each implant and within each surface group. The Mann-Whitney ^19^ ^,^ ^57^ ^,^ ^62^ ^,^ ^63^, Wilcoxon ^43^ ^,^ ^59^ and Kruskall-Wallis ^31^ tests were used to examine differences among the groups and surfaces. Statistical significance was established at the level of 5% (p < 0.05).

### Clinical Observations

In total, 123 micro-implants with 7 different surfaces were inserted, fifteen showed mobility during removal, and were considered lost. Among them, 7 (that is almost 50% of the micro-implants lost) had cpTi surfaces and were removed from the posterior region of the maxilla. Five implants (in smoker patients) were lost, all of them from the posterior region of the maxilla ^62^ ^,^ ^63^. The micro-implants that did not osseointegrate were not included in the histometric evaluations, except in one study ^19^, in which these implants that failed were included with 0% (zero).

Only the micro-implants inserted in smoker patients ^62^ ^,^ ^63^ showed marginal bone resorption. When compared macroscopically, after obtaining the biopsies by using trephine burs, the micro-implants of the Test Groups ^19^ ^,^ ^43^ ^,^ ^57^ ^,^ ^58^ showed a larger quantity of bone tissue.

### Histomorphometry

The data relative to bone density restricted to the threads and close to the implant surfaces were shown only in the studies ^19^ ^,^ ^31^ ^,^ ^43^ ^,^ ^57^ ^,^ ^58^ ^,^ ^59^ ^,^ ^60^ ^,^ ^61^. In this section, only bone-implant contact (BIC) was presented. The characteristics and data of each study are summarized in Box 1.

The peri-implant bone tissue that surrounded the micro-implants showed histological signs of normality for all the surfaces. In most cases, the pristine bone tissue was lamellar and compact, had numerous osteocytes in their gaps, and interlaced with areas of mineralized bone tissue, while the neoformed bone tissue presented at the bone-implant interface showed various stages of maturation and remodeling, with this characteristic being less evident in the micro-implants with Ticp surfaces.

In some cases, there were osteoblasts connected to the neoformed bone tissue, suggesting progressive bone formation. Between the micro-implant threads, a lower level of neoformed bone tissue apposition was found. However, this tissue showed signs of immaturity. Some micro-implants with cpTi surfaces showed discontinuity between the thin bone trabeculae localized at the receptor site and the implant surface. However, irrespective of the type of surface treatment, the other micro-implants showed a thin layer of trabecular bone tissue interposed between the pristine bone and implant surface. In some slides, the area around the cpTi surface showed a thin layer of dense connective tissue between the bone-implant interface. As a complement, some sections of the treated surfaces, mainly on the airborne abraded surfaces treated with acids and the sintered surface, showed inflammatory cells (lymphocytes, macrophages, and giant cells) next to the peri-implant soft tissue, very close to the implant surface (s 3 and 4).

**Figure 3:**
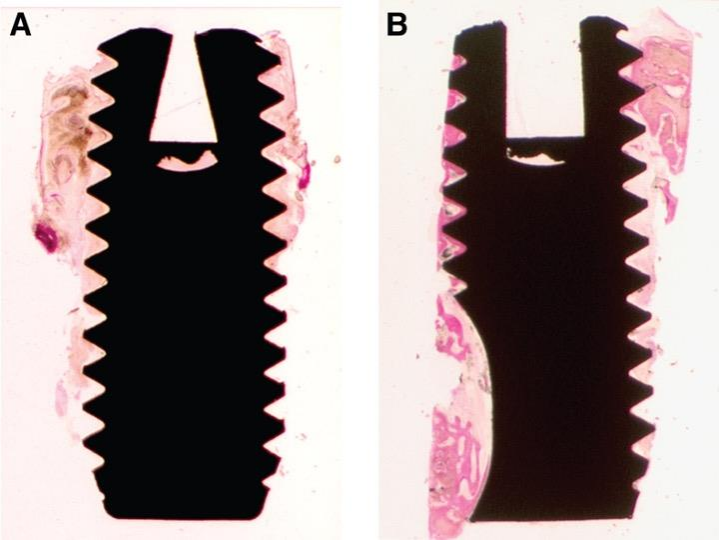
Histologic ground section of the microimplants presented in Figure 1. A) The machined surface depicted newly formed bone, although there is a lack of connecting bridges between new bone trabeculae and the machined surface. B) Ground section of the sandblasted surface presenting newly formed bone which exhibited early stages of maturation and remodeling. (Basic fuchsin and toluidine blue staining; original magnification: A and B, x12.) (Grassi et al. *J. Periodontol* 2006; License number 5800330837972)

**Box 1:**
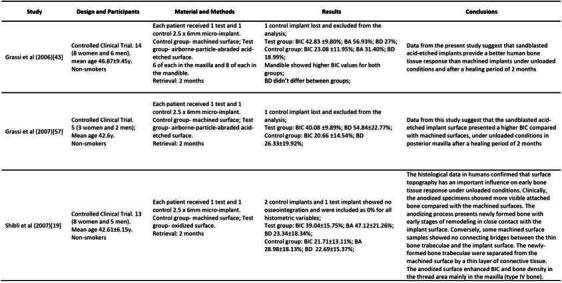
Characteristics and data of each included study. (BIC-Bone to implant contact; BA-bone area within the threads; BD-bone density; Oi- Osteocyte index; SEM- Scanning Electron Microscopy)

**Box 1:**
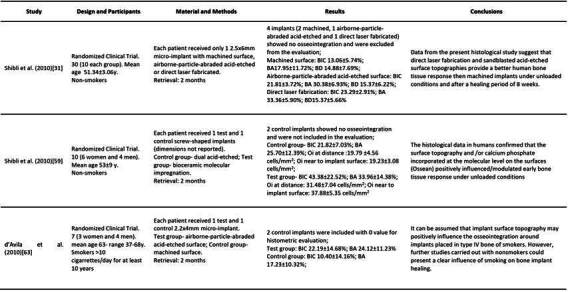
Continuation

**Box 1:**
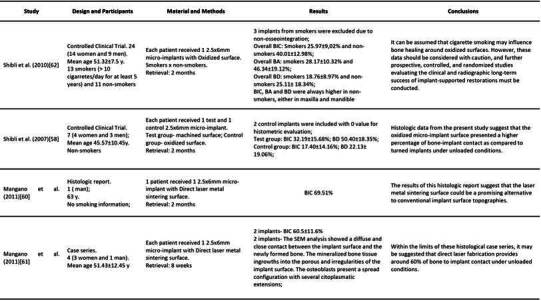
Continuation

**Figure 4:**
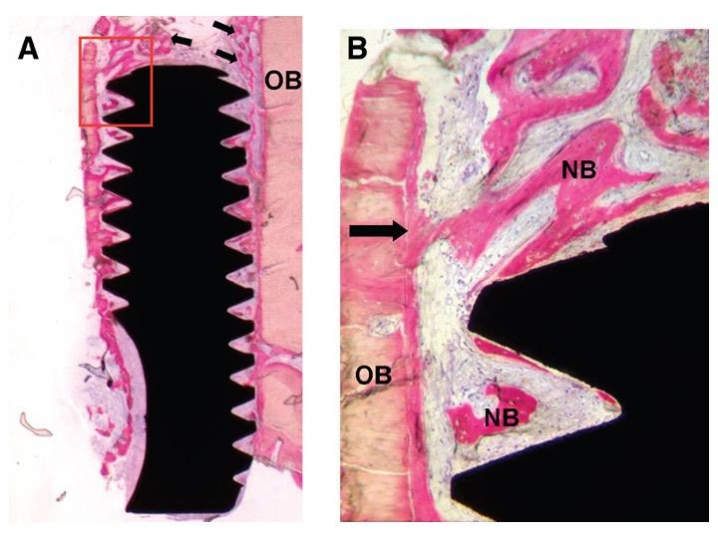
Histologic ground section of a microimplant with sandblasted surface retrieved from the mandible. A) The old bone (OB) was mostly lamellar and compact, and numerous osteocytes were present in the lacunae, although areas of new bone could be distinguished (arrows). B) Magnification of boxed area in A. There is a connecting bridge between the old bone (OB) and the thin new bone (NB) as indicated by the arrow. A minor apposition of new bone is depicted in close contact with the implant surface. (Basic fuchsin and toluidine blue staining; original magnification: A, x12; B, x200.) (Grassi et al. *J. Periodontol* 2006; License number 5800330837972)

**Figure 5:**
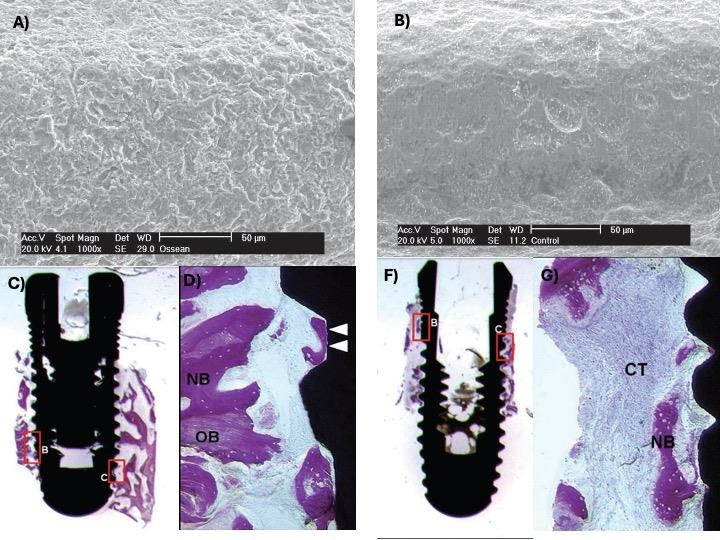
A) Scanning electron microphotograph of the implant surface topographies evaluated, Ossean® and B) dual acid-etched surface; C) Histologic ground section of Ossean® implant. The old bone (OB) was mostly lamellar; D) Larger magnification of the lateral frame areas in the section shown in C. Apposition of new bone (NB) is depicted in close contact (arrowhead) with the implant surface. Reversal lines showing the limits between OB and new bone (NB) (basic fuchsin and toluidine blue staining, original magnification x200). E) Histological ground section of the dual acid-etched surface after 2 months of healing depicting the newly formed bone showing early maturing and remodeling stages. Note the lack of connecting bridges between the new bone trabeculae and the implant surface (basic fuchsin and toluidine blue staining, original x12 magnification); F)A larger magnification of the lateral frame areas in the section shown in E). The newer bone (NB) tissue shows no contact with the implant surface with presence of connective tissue (CT) (basic fuchsin and toluidine blue staining, original x200 magnification). (Shibli et al. *Clin Implant Dent Rel Res* 2010; License number 5800330323137)

The micro-implants showed several levels of BIC%. Although the sizes of the micro-implants differed among them, the measurements were presented in percentages, thereby facilitating comparison. On average, the cpTi surface had a BIC% of 21%, while the airborne abraded surfaces treated with acids showed 38.8% ^43^ ^,^ ^57^. Whereas the anodized surfaces ^19^ ^,^ ^58^ had a mean BIC% value of 38.84%; this percentage was lower than the 43% found for the surface impregnated with CaP, and higher than that found for the surface treated with double acid attack (21.82%) ^59^ (Figure 5)

**Figure 6:**
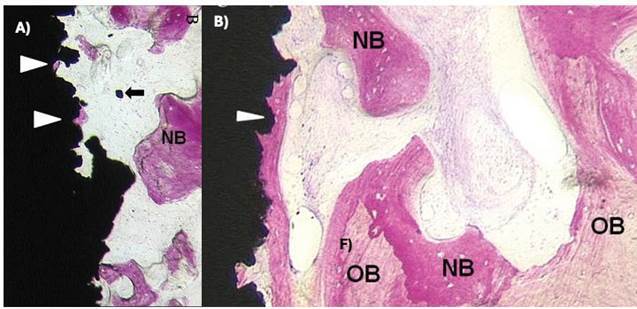
Ground section of the direct laser fabrication surface (DLF) presenting newly-formed bone exhibited early stages of maturation and remodelation. A) A thin layer of bone tissue (NB) in direct contact with DFL topography (arrowhead) suggests osteogenesis of contact. Particle inclusion could be detected (arrow; Basic fuchsin and toluidine blue staining, original magnification x200); (B) Reversal lines separate the newly formed bone (NB) and pristine bone (OB). The Arrowhead depicts the contact osteogenesis next to the implant surface. (Shibli et al. *J Biomed Mat Res A* 2010; License number 5800330712013)

The data obtained in one study ^31^ were very interesting. The BIC% values of the sintered, cpTi and airborne abraded surfaces subsequently treated with acids were lower when compared with the results of other studies ^43^ ^,^ ^57^ (Figure 6). Although the surfaces were the same, the micro-implants were inserted in the posterior region of the maxilla, close to the maxillary tuberosity - an area with predominantly type IV bone ^31^. For example, the mean values of the Ticp surface and surface that was airborne particle abraded and treated with acids were BIC% 13.06% and 21.81% compared with the mean BIC% values of 23.08% and 38.383% respectively, obtained ^43^. The sintered surface had a BIC% value similar to that obtained for the surface that was airborne particle abraded and treated with acids (23.29%; p>0.05), with both values being higher than the mean value of the cpTi surface.

Whereas one study ^61^, by means of superimposition of images, demonstrated a well-delimited structure of calcium (Ca) and phosphorous (P), components of bone tissue, on the titanium (Ti) surface, suggesting bone neoformation on - and mainly within - the entwined canals and pores of the sintered titanium surface.

**Figure 7:**
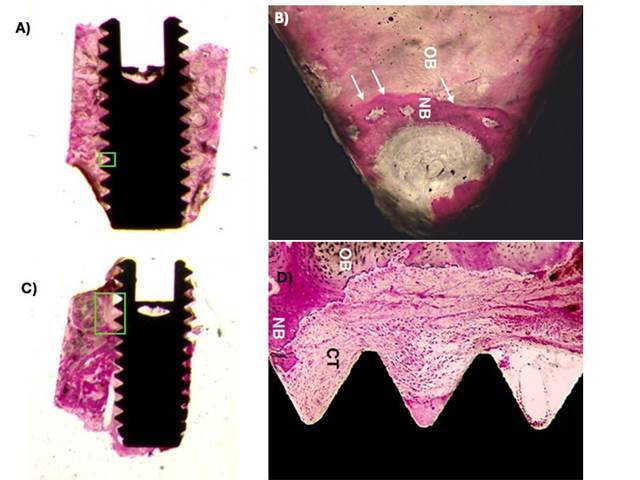
A) Histological ground section of the micro-implant retrieved after 2 months of healing from a posterior mandible of non-smoker depicting the newly formed bone showing early maturing and remodeling stages. (Basic fuchsin and toluidine blue staining, original x12 magnification); (b) higher power view of the section shown in A). The arrows show the reversal lines between newly formed bone (NB) and the older bone (OB) tissue. The newer bone tissue shows direct contact with the sandblasted acid-etched surface. (Basic fuchsin and toluidine blue staining, original 1009 magnification); C) Histological ground section of the micro-implant retrieved after 8 weeks of healing from a posterior maxillae of smoker depicting the newly formed bone showing early maturing and remodeling stages. (Basic fuchsin and toluidine blue staining, original 12x magnification); D) higher power view of the lateral frame area in the section shown in C). The newly formed bone (NB) tissue shows areas of direct contact with the sandblasted acid-etched surface, although some areas there are also a lack of connecting gap and connective tissue (CT) are presented between newly formed bone (NB) and implant surface. CT was loose with scattered inflammatory cells. (Basic fuchsin and toluidine blue staining, original 200x magnification). (Bezerra et al. *Clin Oral Implants Res* 2015; License number 5800330520563)

The histological characteristics of the micro-implants placed in non-smokers from a specific study ^62^ were similar to those observed in some non-smoker’s studies ^19^ ^,^ ^58^: signs of normality of peri-implant bone tissue, with bone neoformation in the initial stages of maturation. However, the implants removed from smoker patients, in addition to a drastic reduction in the quantity of neoformed bone tissue, showed the presence of inflammatory infiltrate and some osteoclasts in the coronal region of the micro-implant. The mean BIC% values were approximately 50% lower when compared with those obtained in nonsmoker patients (p<0.05). The impact of the surface of micro-implants inserted in smoker patients was drastic: the BIC% values were more than twice the value on the treated surface when compared with values on the cpTi surface (p<0.001) ^63^ (Figure 7).

## Discussion

In this study, the surface of micro-implants of Group cpTi had a lower percentage of bone-implant contact when compared with the surface of implants that received some kind of treatment. The topographies produced on the cpTi surface by airborne particle abrasion and subsequent treatment with a mixture of acids had a geometry that could have worked as a restriction on the cellular component of the cytoskeleton of the osteoblast-like cells involved in cell dispersion and locomotion ^66^ ^,^ ^67^. Moreover, Mangano et al. ^61^ suggested that the canals and pores obtained by the fusion and annealment of the titanium particles submitted to fusion and subsequently treated with organic acids could potentiate the previously described effects, thereby increasing osteocondution close to the implant surfaces. As a complement, this characteristic could also explain the increase and speed with which integration of the neoformed bone tissue occurred at the bone-implant interface, similar to the process observed around surfaces with more complex topographies, such as those that were coated with plasma or hydroxyapatite ^30^ ^,^ ^40^ ^,^ ^44^ ^,^ ^67^.

All the micro-implants that were clinically s Box, irrespective of the type of surface, showed some degree of bone neoformation at the bone-implant interface. However, a thin layer of neoformed bone tissue covered a relatively low portion of the micro-implant threads of Group cpTi. This data suggested that the osteoblasts were not able to synthetize enough bone matrix on cpTi surface topography, making it reduced the bone-implant contact to occur by means of direct osteogenesis or contact, differently from the direct contact or osteogenesis observed in various degrees on the other surfaces ^68^ ^,^ ^69^. These data were ratified by the histological findings of the present study. On the surface of micro-implants that received some kind of treatment, it was possible to observe the presence of osteoblasts in direct contact with the implant surface. The data of the present study also suggested that the surface topography was of the utmost importance to the bone tissue response, at least in conditions in which the implants were not submitted to occlusal loading ^7^ ^,^ ^38^ ^,^ ^39^ ^,^ ^40^ ^,^ ^42^ ^,^ ^46^ ^,^ ^70^. Some investigators have also demonstrated that the proliferation and differentiation of osteoblast-like cells are increased by means of the roughness of the implant surface ^37^ ^,^ ^71^ ^,^ ^72^ and by their hydrophilicity ^46^ ^,^ ^73^, ratifying the data of our studies ^19^ ^,^ ^58^.

The healing process begins just after the implant is inserted, with the formation of a blood clot on the implant surface, originating in a thin layer of fibrin ^68^ ^,^ ^69^ ^,^ ^74^ ^,^ ^75^ ^,^ ^76^. The implants of Group cpTi ^19^ ^,^ ^31^ ^,^ ^43^ ^,^ ^57^ ^,^ ^58^ showed low bone density formed between the threads of the micro-implants. This phenomenon may be explained by greater retention of the fibrin network, and consequently, a larger quantity of undifferentiated mesenchymal cells for later activation into osteoblasts.

Smoking negatively influenced these healing events ^62^ ^,^ ^63^ by reducing bone-to-implant contact. The peri-implant mineralization process involves a cascade that activates the synthesis and production of proteins, growth factors, cytokines, and angiogenic stimulators that coordinate the restoration of mechanical stability of bone tissue. Although the impact of cigarettes on human health is notorious, in the case of bone biology, the exact mechanism by which smoking acts on peri-implant bone tissue has not yet been completely elucidated. This is mainly due to the large quantity of toxic substances present in the composition of cigarettes. For example, the expression of collagen type I, alkaline phosphatase, and osteocalcin regulate the mineralization of bone tissue by means of calcium deposition, which is affected by nicotine ^77^.

Smoking is also associated with an increase in oxidant substances and a reduction in vitamins, which is correlated with bone loss in women at the stage of menopause. Nicotine, carbon monoxide and cotinine may affect these cell mechanisms. Vasoconstriction reduces the flow of blood and nutrients to the peri-implant site, inhibiting the proliferation of blood cells responsible for the differentiation of mesenchymal cells, which associated with the lack of oxygen, resulted in tissue hypoxia and retarded the process of osseointegration, resulting in the low osseointegration indexes ^62^.

It should also be pointed out that the profilometric style of the treated surfaces, particularly those airborne particles abraded and treated with acids, as well as those sintered, may also provide a better condition for stabilizing the blood clot, thereby facilitating bone healing on the surface of the implant ^13^ ^,^ ^44^. This fact would also explain the significant differences between the treated and cpTi surfaces ^63^.

Some studies have shown that the implant's surface topography could affect not only the osteoblast's gene expression but also the differentiation of the cells into osteoblasts ^73^ ^,^ ^76^ ^,^ ^78^. Furthermore, these authors suggested that the interaction of cells with the components of the extracellular matrix and the organization of the cytoskeleton-associated with the implant topography could influence the cell’s gene expression. Consequently, this would increase the formation of a bone matrix in close contact with the implant surface, even in smoker patients.

The data obtained for Group cpTi ratified the previous findings ^15^ ^,^ ^16^ ^,^ ^17^ ^,^ ^79^ ^,^ ^80^, which affirmed that this surface did not provide a strong anchorage for the bone tissue, particularly in sites with low-density bone, such as those localized in the posterior region of the maxilla, grafted areas and in smoker patients. These observations explained the high rate of losses reported in some investigations ^15^ ^,^ ^16^ ^,^ ^17^. Therefore, the surface topography may be considered one of the most important factors capable of influencing the long duration or survival of the implant inserted in type IV bone.

Moreover, other studies have demonstrated that the anchorage of machined implants is time-dependent; that is, they need a longer healing time than rough surface implants ^81^ ^,^ ^82^. Nevertheless, some studies in humans ^38^ ^,^ ^42^, using a methodology similar to that used in our study, evaluated the bone-implant contact of implants with machined surfaces inserted in human maxillae, and showed a percentage of bone-implant contact of approximately 9% and 13% respectively, after a healing period of 5 to 6 months. These values were lower when compared with the 19% bone-implant contact showed by Trisi et al. ^32^, who evaluated micro-implants inserted in the posterior region of the maxilla of humans after 60 days of healing. This suggested that the machined surface depends far more on the bone density of the receptor site than on the osteoconductive property of its surface. On the other hand, roughness surface is less impacted by the bone density than cpTi surfaces, as roughness surface present contact osteogenesis as mentioned before. Therefore, the importance of the result of this study with treated surface, in view of the mean BIC% and Bone Density values, in comparison with those of Group cpTi, once again confirmed the possibility of a reduction in the healing period in certain situations. This agrees with some clinical studies with more than a 3-year follow-up period, with a success rate higher than 94% ^15^ ^,^ ^17^ ^,^ ^20^. Or even in an extrapolation of the clinical results, the use of anodized surfaces or those that were airborne particle abraded and subsequently treated with acids in smoker patients was able to reduce early implant losses and marginal bone losses ^58^.

Another important point concerns the experimental design of this study: histological and histometric evaluation of human peri-implant bone. Lang et al. ^46^ showed that although there may exist some kind of correlation between the findings in animal and in human models, factors inherent to human beings may change the biological sequence and, for example, increase the necessary period for bone healing. Nevertheless, the use of implants with reduced dimensions (micro-implants) provided an excellent opportunity to evaluate the behavior of human bone tissue in response to different surfaces of osseointegrated implants. This was, considering the difficulty of obtaining implants that have been removed from individuals, in which a systematic evaluation could be made of the impact of these microstructures on osseointegration. 

## Conclusions

Based on the results of the articles of this narrative review, it could be suggested that the implant surface influences the bone response in the initial periods of healing when not being submitted to masticatory load. Moreover, within the limits of the results:

- Treated surfaces, irrespective of the type of treatment, showed better histometric results when compared with the Ticp surface.
- Smoking habit reduced the bone-implant contact around anodized implants.
- In smoker patients, the Surfaces of implants that were airborne particle abraded and treated with acids showed higher mean bone-implant contact values when compared with the Ticp surface.

Finally, this review is limited to studies conducted by a single research group employing a single methodology. Future research should include additional histologic studies utilizing proteomics to identify potential markers present in peri-implant bone tissue around various implant surfaces. Moreover, clinical studies with long-term follow-up are essential to fully understand the impact of implant topography on clinical outcomes. These advancements are crucial for a comprehensive understanding of the subject.
